# Similar incidence of coronavirus disease 2019 (COVID-19) in patients with rheumatic diseases with and without hydroxychloroquine therapy

**DOI:** 10.1371/journal.pone.0249036

**Published:** 2021-04-08

**Authors:** Juan Macías, Paz González-Moreno, Esther Sánchez-García, Ramón Morillo-Verdugo, José J. Pérez-Venegas, Ana Pinilla, MªMar Macho, MªVictoria Martínez, Alejandro González-Serna, Anaïs Corma, Luis M. Real, Juan A. Pineda

**Affiliations:** 1 Infectious Diseases and Microbiology Unit, Hospital Universitario Virgen de Valme, Seville, Spain; 2 Rheumatology Unit, Hospital Universitario Virgen Macarena, Seville, Spain; 3 Internal Medicine Service, Hospital Universitario Virgen de Valme, Seville, Spain; 4 Pharmacy, Hospital Universitario Virgen de Valme, Seville, Spain; Instituto Nacional de Ciencias Medicas y Nutricion Salvador Zubiran, MEXICO

## Abstract

**Background:**

Hydroxychloroquine is not efficacious as post-exposure prophylaxis against coronavirus disease 2019 (COVID-19). It is not known whether as pre-exposure prophylaxis it may prevent COVID-19.

**Objective:**

To compare the incidence of COVID-19 in Spanish patients with autoimmune rheumatic diseases treated with and without hydroxychloroquine.

**Patients and methods:**

Retrospective electronic record review, from February 27^th^ to June 21^st^, 2020, of patients with autoimmune inflammatory diseases followed at two academic tertiary care hospitals in Seville, Spain. The cumulative incidence of confirmed COVID-19, by PCR or serology, was compared between patients with and without hydroxychloroquine as part of their treatment of autoimmune inflammatory diseases.

**Results:**

Among 722 included patients, 290 (40%) were receiving hydroxychloroquine. During the seventeen-week study period, 10 (3.4% [95% CI: 1.7%-6.7%] cases of COVID-19 were registered among patients with hydroxychloroquine and 13 (3.0% [1.6%-5.1%]) (p = 0.565) in those without hydroxychloroquine. COVID-19 was diagnosed by PCR in four (1.4%, 95% CI 0.38%-3.5%) subject with hydroxychloroquine and six (1.4%, 95% CI 0.5%-3.0%) without hydroxychloroquine (p = 0.697). Three patients on hydroxychloroquine and four patients without hydroxychloroquine were admitted to the hospital, none of them required to be transferred to the intensive care unit and no patient died during the episode.

**Conclusions:**

The incidence and severity of COVID-19 among patients with autoimmune rheumatic diseases with and without hydroxychloroquine was not significantly different.

## Introduction

Coronavirus disease 2019 (COVID-19) pandemic is currently a health emergency which has caused 935.767 deaths worldwide in four months since 31 December 2019 and as of 16 September 2020 [1]. Because of this, finding effective therapy and prophylactic strategies has become a maximum priority [2]. Hydroxychloroquine (HCQ) has shown to be active against severe acute respiratory syndrome coronavirus 2 (SARS-CoV-2) *in vitro* by inhibiting several steps of the viral replication cycle, including some of the earliest ones, as the fusion to cell membrane [3]. However, in an underpowered clinical trial, due to the lack of enough eligible patients to enroll, HCQ led to a similar probability of virus elimination to the standard of care arm among patients with mild to moderate COVID-19 [4]. In addition, the HCQ arm was interrupted in large clinical trials after interim analyses showing no beneficial effect on COVID-19 patients when compared to standard of care [5, 6]. Because of these, after some controversy on the clinical effectivity of HCQ [7, 8], this drug is finally not recommended to treat COVID-19.

Due to the *in vitro* activity of HCQ against SARS-CoV-2, using this drug was proposed as a rational strategy for post-exposure prophylaxis [9]. Thus, at least 11 clinical trials aimed to test this hypothesis are currently ongoing, and three have been completed [10]. A study reported that post-exposure HQC provided to inpatients and healthcare professionals with documented contact with an index COVID-19 case in a long-term care hospital was safe and associated with no secondary COVID-19 cases [11]. However, in a double-blind, placebo-controlled trial, high dose of HCQ did not prevent illness compatible with COVID-19 when used within 4 days after high-risk or moderate-risk exposure [12]. HCQ is commonly used as a part of the therapy of several autoimmune inflammatory diseases, such as rheumatoid arthritis (RA) or systemic lupus erythematous (SLE). In this setting, HCQ is administered continuously at lower doses than those reported for COVID-19 treatment or post-exposure prophylaxis. If HCQ was effective as pre-exposure prophylaxis of SARS-CoV-2, a reduced incidence of COVID-19 could be expected in patients with autoimmune inflammatory diseases receiving treatment with this drug. Data on this issue may provide us with information on the potential of HCQ therapy as a prophylactic strategy, which may help to design pre-exposure prophylaxis clinical trials. Because of this, in this study we aimed to compare the incidence of COVID-19 in Spanish patients with autoimmune rheumatic diseases treated with HCQ and without HCQ therapy, during the first wave of the pandemic in the country.

## Patients and methods

This was a cross-sectional retrospective study, where patients with autoimmune inflammatory disorders in which HCQ is commonly used, were included. All patients attended by two specialists at the Rheumatology Unit of Virgen Macarena University hospital and by one specialist at the Internal Medicine Unit of Virgen de Valme University Hospital, both in Seville (Spain), during six months before the study period were identified. Patients who died before December 31^st^, 2019 were excluded.

In these patients with autoimmune inflammatory disorders, we evaluated the cases diagnosed with COVID-19 from February 27^th^ to June 21^st^, 2020. Patients were contacted by telephone to evaluate whether they were exposed to known COVID-19 cases. The first COVID-19 was identified in Seville on February 27^th^, and the end of the state of alarm was on June 21^st^. During the majority of this period of time, SARS-CoV-2 transmission in Spain was regarded as community transmission, i.e. most cases could not be traced within a chain of transmission.

To evaluate the number of COVID-19 cases among the study patients, we searched for episodes of attendance to hospitals and primary care because of COVID-19 related symptoms in the shared electronic medical record of the hospitals of the Andalusian Health Service. COVID-19 cases were defined as those with positive results of SARS-CoV-2 PCR (cobas^®^ SARS-CoV-2 Test, Roche Diagnostics) in nasopharynx swab or serum antibody tests (Elecsys® Anti-SARS-CoV-2, Roche Diagnostics; Orient Gene Biotech COVID-19 IgG/IgM Rapid Test Cassette, Zhejiang Orient Gene Biotech). Probable COVID-19 cases, according to the WHO criteria, were identified and, whenever possible, serum antibody tests were determined for confirmation.

The drugs prescribed to patients were identified using the shared electronic medical record of the hospitals of the Andalusian Health Service and the electronic Pharmacy records. Patients taking HCQ during the full period of observation were classified as HCQ group.

In the statistical analysis, continuous variables were compared by the Mann-Whitney U test and the categorical ones by the Fisher test. For the main rates, percentages and 95% confidence intervals (95% CI) were estimated. Statistical analyses were conducted using the package STATA 16.0 StataCorp, College Station, TX, USA. The sample size calculations were carried out and approved before the study started (S1 File).

The study was designed and performed according to the Helsinki declaration and approved by the Ethics Committee of the Valme University Hospital (Seville, Spain). Informed consent was waived by the Ethics Committee of the Valme University Hospital due to the observational retrospective design of the study, and because data was anonymized. Telemedicine, and specifically phone calls, were part of the routine clinical assessment during the period of study. Our Ethics Committee did not consider those routine phone calls and the clinical information extracted from them should be considered a prospective intervention.

## Results

Seven hundred and twenty-two patients were studied. Two hundred and ninety (40%) of them were receiving HCQ. The main features of the study population, including those who were on HCQ therapy and those who were not, are shown in Table 1.

**Table 1:** Features of the study patients, according to they were on HCQ or not.

Characteristic	HCQ (n = 290)	No HCQ (n = 432)	p value
Median (Q1-Q3) age, years	56 (45–65)	58 (48–68)	0.140
Male sex, n (%)	42 (16)	82 (21)	0.123
Rheumatic disease, n (%)			<0.001
RA	144 (50)	323 (75)	
SLE	83 (30)	11 (2.5)	
Other	60 (21)	98 (23)	
Immunosuppressive treatment, n (%)	169 (58)	321 (74)	<0.001
Anti-TNF drugs, n (%)	3 (1)	54 (13)	<0.001
Corticosteroids, n (%)	112 (39)	149 (35)	0.257
Latest median (Q1-Q3) CRP, mg/dl before diagnosis	3.0 (1.0–4.5)	3.6 (1.2–5.3)	0.802
COVID-19 diagnosis, n (%)	10 (3.4)	13 (3)	0.565
Confirmed by PCR	4 (40)	6 (46)	
Confirmed by serology	6 (60)	7 (54)	
Admissions, n/N (%)	3/10 (30)	4/13 (31)	1.0

HCQ: Hydroxychloroquine RA: Rheumatoid arthritis; SLE: Systemic lupus erythematosus; TNF: Tumor necrosis factor alpha. CRP: C-reactive protein. Other: Includes mixed connective tissue disease, scleroderma, spondylarthritis, undifferentiated polyarthritis, reactive arthritis, sarcoidosis, Sjogren syndrome, antiphospholipid syndrome, dermatomyositis, and Behçet disease.

During the seventeen-week study period, 36 (12.4%) patients on HCQ and 49 (11.3%) patients without HCQ were tested because of symptoms suggestive of COVID-19. Among patients with suspected COVID-19, SARS-CoV-2 PCR was applied in 14 (3.8%) of 36 patients on HCQ and 21 (4.3%) of 49 patients without HCQ. The rest of the patients were tested with serology. Overall, 10 (3.4% [95% CI: 1.7%-6.7%] cases of COVID-19 were registered among patients treated with HCQ and 13 (3.0% [1.6%-5.1%]) (p = 0.565) in the group of patients who were not receiving this drug. COVID-19 was diagnosed by PCR in four (1.4%, 95% CI 0.38%-3.5%) subject treated with HCQ and six (1.4%, 95% CI 0.5%-3.0%) without this drug (p = 0.697) (Table 1). A previous contact with a case with COVID-19 was reported by four (36%) patients on HCQ and five (38%) patients without HCQ (p = 1.0). Three patients on HCQ and four patients without HCQ were admitted to the hospital, none of them required to be transferred to the intensive care unit (ICU) and no patient died during the episode. The length of stay was 24 (range: 16–31) days for patients on HCQ and 21.5 (range: 14–34) days.

Immunosuppressive treatment includes: abatacept, azathioprine, baricitinib, belimumab, corticosteroids, cyclophosphamide, leflunomide, methotrexate, mycophenolate, tacrolimus, tofacininib, anti-CD20, anti-IL1, anti-IL6, anti-IL12 and anti-IL23 and anti-IL17 drugs.

## Discussion

In the present study, the incidence of confirmed COVID-19 among patients with autoimmune rheumatic diseases on HCQ treatment was not significantly different of that observed in patients not receiving HCQ. In addition, the severity of COVID-19, as measured by the rate of admission or ICU requirement, was similar in both groups. These results suggest that continuous use of HCQ during the pandemic of COVID-19 did not prevent SARS-CoV-2 infections.

Patients with immune-mediated inflammatory diseases are a population with particular features, that includes frequent use of biologic agents, other immunomodulatory drugs or both. The use of immunosuppressive drugs could increase the incidence of SARS-CoV-2 infection or worsen the outcome of COVID-19. However, preliminary data from a case-series in New York City suggested that the use of these drugs is not associated with a different clinical profile in COVID-19 [13]. In another study, the risk of admission for COVID-19 was higher for people with rheumatological disease receiving corticosteroids, but the odds of hospitalization among those on biological agents were not increased [14]. Moreover, HCQ exposure was not related with a lower risk of admission in that report [14]. These results contrast with a study that showed that patients with autoimmune rheumatic diseases had a higher rate of COVID-19 than their family members living in the same household during the outbreak in Hubei [15]. In that study, the likelihood of COVID-19 was lower for patients taking HCQ. However, as the authors acknowledged, the number of patients with exposure to HCQ was small and the lower COVID-19 incidence observed in comparison with patients taking other immunosuppressive medications should be interpreted with caution [15]. In our study, during the period of observation, in the province of Seville, an area with 1940000 inhabitants, 3178 confirmed COVID-19 cases were reported [16]. This yields an overall incidence of 0.164% (95% CI 0.16%-0.17%) cases. This figure is inside the 95% CI of confirmed COVID-19 found herein both in patients taking HCQ and in those who were not on this therapy. This suggests that the incidence of confirmed SARS-CoV-2 infection among patients with autoimmune rheumatic diseases, with or without HCQ, is similar to the incidence of SARS-CoV-2 infection in the general population.

This study has a few limitations. First, shortage of diagnostic kits and health care system collapse in Spain have led to the fact than many cases of COVID-19 were not able to be confirmed during the first weeks of the outbreak. In addition, data come only from hospital registers and mild cases could have been seen only in primary care; likewise, most asymptomatic cases would have gone unnoticed. Due to all these reasons, a small number of COVID-19 cases was detected in both groups. However, even having omitted patients, these data show that incidence of COVID-19 in patients treated with HCQ would be far from zero. Therefore, HCQ would not be an ideal therapy for pre-exposure prophylaxis of SARS-CoV-2 infection.

In summary, HCQ as post-exposure prophylaxis against SARS-CoV-2 infection was not effective in controlled clinical trials [12]. Whether pre-exposure prophylaxis would be effective is a separate question. However, according to our results, other strategies should be designed to be tested, since continuous administration of HCQ, as in pre-exposure prophylaxis, does not seem to reduce the incidence of SARS-CoV-2 infection.

## Supporting information

S1 File (DOCX)
