# Supplementary material for: Similar incidence of coronavirus disease 2019 (COVID-19) in patients with rheumatic diseases with and without hydroxychloroquine therapy
Source: PLoS One. 2021 Apr 8;16(4):e0249036. doi: 10.1371/journal.pone.0249036 (PMC8031374; doi:10.1371/journal.pone.0249036)
Supplement: S1 File — (DOCX) [file pone.0249036.s001.docx]

**Original simple size calculation**

As of March 18, 191,726 cases of COVID-19 and 20,043 (10.5%) deaths have been reported in Spain. The real mortality rate is estimated at around 1%. Therefore, the real epidemic in Spain would correspond to 10 times what was reported, so that it could be estimated that 1,917,260 subjects currently have COVID-19. For a Spanish population of 47,000,000 inhabitants, the real prevalence of COVID-19 in the general population would be 4%. To achieve a power of 80%, with a significance level of 5%, and assuming that the frequency of COVID-19 of 5% in the group without HCQ, similar to the general population, and that in the group with HCQ it will be 1%, with a proportion of patients without HCQ of 60%, it will be necessary to include 373 subjects without HCQ and 249 subjects with HCQ.

We finally recruited 432 patients without HCQ and 290 with HCQ. The pre-planned sample size was reached.

The seroprevalence in a nationwide survey in Spain was 5% (95% CI 4.7-5.4) (Pollán M, et al. Prevalence of SARS-CoV-2 in Spain (ENE-COVID): a nationwide, population-based seroepidemiological study. Lancet. 2020;396:535-544.). Thus, our estimation of a frequency of 5% in the group without HCQ, similar to the general population, was later supported by data. The 95% CI of confirmed COVID-19 found in our study of both the HCQ group and the non-HCQ group are within the 95% CI of the seroprevalence figure. Therefore, the rate of confirmed COVID-19 in both groups lies within the expected value for Spanish general population.
